# MicroRNA-210 downregulates TET2 and contributes to inflammatory response in neonatal hypoxic-ischemic brain injury

**DOI:** 10.1186/s12974-020-02068-w

**Published:** 2021-01-05

**Authors:** Qingyi Ma, Chiranjib Dasgupta, Guofang Shen, Yong Li, Lubo Zhang

**Affiliations:** The Lawrence D. Longo Center for Perinatal Biology, Department of Basic Sciences, Loma Linda University School of Medicine, Loma Linda, CA 92350 USA

**Keywords:** Neonatal hypoxia-ischemia, MicroRNA-210, The ten eleven translocation (TET) methylcytosine dioxygenase 2, Acetyl-p65, Pro-inflammatory cytokines, BV2 mouse microglia cell line, Liposaccharide (LPS)

## Abstract

**Background:**

Neonatal hypoxic-ischemic (HI) brain injury is a leading cause of acute mortality and chronic disability in newborns. Our previous studies demonstrated that HI insult significantly increased microRNA-210 (miR-210) in the brain of rat pups and inhibition of brain endogenous miR-210 by its inhibitor (LNA) provided neuroprotective effect in HI-induced brain injury. However, the molecular mechanisms underpinning this neuroprotection remain unclear.

**Methods:**

We made a neonatal HI brain injury model in mouse pups of postnatal day 7 to uncover the mechanism of miR-210 in targeting the ten eleven translocation (TET) methylcytosine dioxygenase 2 that is a transcriptional suppressor of pro-inflammatory cytokine genes in the neonatal brain. TET2 silencing RNA was used to evaluate the role of TET2 in the neonatal HI-induced pro-inflammatory response and brain injury. MiR-210 mimic and inhibitor (LNA) were delivered into the brain of mouse pups to study the regulation of miR-210 on the expression of TET2. Luciferase reporter gene assay was performed to validate the direct binding of miR-210 to the 3′ untranslated region of the TET2 transcript. Furthermore, BV2 mouse microglia cell line was employed to confirm the role of miR-210-TET2 axis in regulating pro-inflammatory response in microglia. Post-assays included chromatin immunoprecipitation (ChIP) assay, co-immunoprecipitation, RT-PCR, brain infarct assay, and neurobehavioral test. Student’s *t* test or one-way ANOVA was used for statistical analysis.

**Results:**

HI insult significantly upregulated miR-210, downregulated TET2 protein abundance, and increased NF-κB subunit p65 acetylation level and its DNA binding capacity to the interleukin 1 beta (IL-1β) promoter in the brain of mouse pups. Inhibition of miR-210 rescued TET2 protein level from HI insult and miR-210 mimic decreased TET2 protein level in the brain of mouse pups, suggesting that TET2 is a functional target of miR-210. The co-immunoprecipitation was performed to reveal the role of TET2 in HI-induced inflammatory response in the neonatal brain. The result showed that TET2 interacted with NF-κB subunit p65 and histone deacetylase 3 (HDAC3), a co-repressor of gene transcription. Furthermore, TET2 knockdown increased transcriptional activity of acetyl-p65 on IL-1β gene in the neonatal brain and enhanced HI-induced upregulation of acetyl-p65 level and pro-inflammatory cytokine expression. Of importance, TET2 knockdown exacerbated brain infarct size and neurological deficits and counteracted the neuroprotective effect of miR-210 inhibition. Finally, the in vitro results demonstrated that the miR-210-TET2 axis regulated pro-inflammatory response in BV2 mouse microglia cell line.

**Conclusions:**

The miR-210-TET2 axis regulates pro-inflammatory cytokine expression in microglia, contributing to neonatal HI brain injury.

## Background

Neonatal hypoxic-ischemic encephalopathy (HIE) is the most common cause of neonatal brain damage triggered by systemic asphyxia, which mainly occurs during the perinatal period. HIE is a leading cause of acute mortality and chronic disability in newborns [1–5] and is associated with short-term medical complications and long-term mental and other neurological disorders with delayed clinical onset [6, 7]. Currently, hypothermia therapy is the standard of care for perinatal HIE, which provides some degree of success in clinical treatment [8–11]. However, the incidence of death and disability remains as high as about 40% of treated infants [12, 13]. Thus, there is an urgent need to advance our knowledge of the molecular mechanisms of neonatal HIE and develop operative therapeutic interventions to improve the outcome of clinical care.

An increasing body of evidence shows that inflammatory response is a critical contributor to the pathophysiology of neonatal hypoxic-ischemic (HI) brain injury [14–17]. The innate immune system is initiated within minutes after cerebral ischemic insult in neonates [18]. HI insult induces the activation of brain resident immune cells and transmigration and infiltration of peripheral immune cells crossing the blood-brain barrier into the brain parenchyma. Subsequently, the release of pro-inflammatory mediators amplifies inflammatory cascades and ultimately results in brain injury [15, 16]. Mounting preclinical evidence supports the significant role of inflammation in the neonatal brain with HI insult [17, 19], and inhibition of the early inflammatory phase in the setting of HI brain injury confers neuroprotection [20, 21]. Moreover, elevated levels of pro-inflammatory cytokines and chemokines have been found in the cerebrospinal fluid and/or blood of infants with HIE, which are associated with adverse neurological outcome and correlate strongly with the likelihood of cerebral palsy [22–24].

It has been increasingly recognized that microRNAs (miRs) contribute to the regulation of both innate and adaptive immune cell function in neurological disorders such as ischemic stroke [25–28]. MiRs are a group of non-coding RNAs with about 21–22 nucleotide long and function in silencing gene expression by binding to the 3′ untranslated region (3′UTR) of the transcripts of target genes [29, 30]. We have demonstrated that inhibition of brain miR-210 with its complementary locked nucleic acid oligonucleotides (miR-210-LNA) suppresses ischemic stroke-induced inflammatory response in the brain of adult mice [31] and also provides neuroprotection in the brain of rat pups after HI insult [32, 33]. However, as the *Master Hypoxamir* [34], the role of miR-210 in the regulation of inflammatory response in neonatal HI brain injury remains largely unknown.

Herein, we uncover a novel mechanism by which miR-210 targets the 3′UTR of TET2 transcript and represses TET2 expression in the neonatal brain in response to HI insult. TET2 orchestrates the expression of pro-inflammatory cytokines through regulating the acetylation levels of NF-κB subunit p65 in the neonatal brain. Furthermore, we demonstrate that TET2 downregulation exaggerates HI-induced brain infarct size and neurological deficits and counteracts the neuroprotection of miR-210 inhibition in neonatal HI brain injury. Additionally, using BV2 microglia, we provide evidence that the miR-210-TET2 axis regulates proinflammatory response in microglia.

## Methods

### Neonatal mouse model of hypoxia-ischemia (HI)

A modified Rice-Vannucci model was produced in 7-day-old (P7) CD1 mouse pups as we previously described [35]. Briefly, mouse pups (Charles River Laboratories) were fully anesthetized with inhalation of 2–3% isoflurane. The right common carotid artery (CCA) in the neck was exposed, double ligated with an 8.0 silk surgical suture, and then cut between two ligation sites. After surgery, pups were recuperated on a heating pad at 37 °C for 1 h and then placed in a hypoxic incubator containing humidified 8% oxygen balanced with 92% nitrogen at 37 °C for 20 min. At the end of hypoxia, pups were returned to their dams for recovery. For the sham group, the right common carotid artery of mouse pup was exposed but without ligation and hypoxia. Mouse pups of mixed males and females were randomly assigned into each experimental group. All procedures and protocols were approved by the Institutional Animal Care and Use Committee of Loma Linda University and followed the guidelines by the National Institutes of Health Guide for the Care and Use of Laboratory Animals.

### Intracerebroventricular (i.c.v.) injection

Compounds with the total volume of 2 μl were stereotaxically injected into the ipsilateral hemisphere of mouse pups intracerebroventricularly (0.8 mm lateral, 1.5 mm below the skull surface) with a flow rate of 0.5 μl/min as described previously [35, 36].

MiR-210-LNA (4103837-102, Exiqon) and LNA scramble control (199006-102, Exiqon) were prepared according to the manufacturer’s instructions. Either miR-210-LNA (100 pmol in 2 μl) or its negative control was delivered into the ipsilateral hemisphere of the brain via i.c.v. injection 24 h before HI insult for TET2 protein level assay or 4 h after HI insult for functional assay. The treatment regimen of miR-210-LNA was determined in our previous studies [32, 33], in which miR-210-LNA with this dose showed neuroprotective effect.

MiR-210 mimic (MSY0000881, Qiagen) and ALLStars negative control (SI03650318, Qiagen) were prepared according to the manufacturer’s instructions. Either miR-210 mimic (100 pmol in 2 μl) or its negative control was injected into the brains of mouse pups, and brain samples were collected 48 h after injection. The treatment regimen of miR-210 mimic was determined in our previous studies [32, 33], in which miR-210 mimic with this dose significantly downregulated the expression of target genes.

Accell mouse TET2 siRNA SMARTPOOL (E-058965, Dharmacon) and negative control siRNA were prepared according to the manufacturer’s instructions. Either TET2 siRNAs (100 pmol in 2 μl) or negative control was administered into the brains of mouse pups via i.c.v. injection. Some animals were sacrificed for TET2 protein abundance assay, and some animals were subjected to HI 48 h after injection.

### Measurement of brain infarct size

Brain infarct size was determined 48 h after HI using 2, 3, 5-triphenyltetrazolium chloride monohydrate (TTC; T8877, Sigma-Aldrich) staining as described previously [35]. Briefly, the brain was isolated from each pup, dissected into coronal sections (2 mm thickness, 4 slices per brain), and immersed into pre-warmed 2% TTC in 0.1 M phosphate-buffered saline (PBS, pH 7.4) at 37 °C for 5 min against light. Sections were washed with PBS and then fixed by 10% formaldehyde overnight. The caudal and the rostral surfaces of each slice were photographed using a digital camera, and the percentage of infarct area (average of both sides) in the ipsilateral hemisphere for each slice was traced and analyzed by NIH Image J software.

### Neurobehavioral assay

The foot-fault test measures the forelimb misplacement on a grid during locomotion. The performance of mouse was videotaped for 5 min or until 50 steps were taken with one forelimb. The total number of steps and times each forelimb fell below the grid was counted by an observer blinded to experimental groups. The percentage of foot-faults for contralateral forelimb and hindlimb to total steps was calculated and presented as previously reported [31]. Wire hanging test tests the forelimb strength of pups, including arm and paw strength. Pups were allowed to grasp a wire string across a stable object and hanged onto the wire with both forepaws. The testing area is over a padded drop zone. The latency to when the animal falls was recorded. The test was performed for three trials [37, 38].

### Cell culture and treatment

BV2 mouse microglia cell line was provided by Dr. Grace Y. Sun (University of Missouri). BV2 cells retain many morphological and functional properties of primary microglia [17]. Cells were cultured in Dulbecco’s modified Eagle’s medium (DMEM; Hyclone Co.) with 10 % fetal bovine serum (FBS; Hyclone Co.), 2 mM L-glutamine, 100 U/ml penicillin, and 100 μg/ml streptomycin (Sigma) in a humidified incubator at 37 °C with 5% CO_2_. To determine the effect of liposaccharide (LPS, Sigma) concentration on microglia stimulation, BV2 cells with 80–90% confluency were incubated with multiple doses (0, 10, 100, or 500 ng/ml) of LPS for 6 h. A total of 100 nM of miR-210 mimic, negative mimic (Qiagen), miR-210-LNA, LNA scramble control (Exiqon), TET2 siRNAs (si. *Tet2*), or control siRNA (si. *Ctrl*, Dharmacon) were used for in vitro transfection. Transfection was performed with HiPerfect transfection reagent (Qiagen) according to the manufacturer’s instructions. BV2 cells were starved in serum-free medium for 2 h before transfection. At 24 h after transfection, cells were stimulated with or without LPS (1 or 500 ng/ml) for 6 h and then collected for assays.

### Measurement of miR-210

The levels of miR-210 were detected as we previously described [32, 33]. Total RNA was extracted using the TRIzol reagent (15596026; Invitrogen). MiR-210 levels were analyzed by miScript II RT kit (218161, Qiagen) and miScript SYBR Green PCR kit (218073, Qiagen) with miScript Primer Assay kit (MS00000644, Qiagen) according to the manufacturer’s instructions. Briefly, 1 μg of template RNA was mixed with reverse-transcription master mix in a final volume of 20 μl and incubated at 37 °C for 60 min, and then the reaction was stopped at 95 °C. Two nanograms of template cDNA was used for miR-210 quantification in a final volume of 25 μl system containing specific primers and QuantiTect SYBR Green PCR master mix according to the manufacturer’s instructions. Primers included miScript Universal Primer, miR-210 miScript Primer Assay, and SNORD61 miScript Primer Assay (MS00033705, Qiagen). PCR was done in triplicate and threshold cycle numbers were averaged for each sample. The relative expression levels of mature miR-210 were calculated using the formula 2(^-ΔΔCt^) and normalized to SNORD61. The change of miR-210 was expressed as fold of normal control.

### qRT-PCR of pro-inflammatory cytokine quantification

Total RNA was subjected to reverse transcription with Superscript III First-Strand Synthesis System (18080051, Invitrogen), following the manufacturer’s instructions. The target gene mRNA abundance was determined with real-time PCR using iQ SYBR Green Supermix (1708880, Bio-Rad) as we previously described [16]. Primers used were listed in Table 1. Real-time PCR was performed in a final volume of 25 μl and each PCR reaction mixture consisted of specific primers and iQ SYBR Green Supermix. PCR was done in triplicate and threshold cycle numbers were averaged for each sample. The relative expression levels were calculated using the formula 2(^-ΔΔCt^) and normalized to actin. The change of mRNA abundance was expressed as fold of normal control.

**Table 1:** Primer sets used for qPCR

	Forward primer (5′-3′)	Backward primer (5′-3′)
TNF-α	CAGCCGATGGGTTGTACCTT	GGCAGCCTTGTCCCTTGA
IL-6	CCACGGCCTTCCCTACTTC	TGGGAGTGGTATCCTCTGTGAA
IL-1β	GAGTGTGGATCCCAAGCAAT	TACCAGTTGGGGAACTCTGC
IFN-γ	TGCTGATGGGAGGAGATGTCT	TGCTGTCTGGCCTGCTGTTA
CCL2	AGGTGTCCCAAAGAAGCTGTAG	AATGTATGTCTGGACCCATTCC
CCL3	TGGAACTGAATGCCTGAGAGT	TAGGAGATGGAGCTATGCAGGT
GAPDH	CGACAGTCAGCCGCATCTT	CCAATACGACCAAATCCGTTG
IL-1β ChIP	TCCCTGGAAATCAAGGGGTGG	TCTGGGTGTGCATCTACGTGCC

### Western blotting

Protein extraction of ipsilateral hemisphere of mouse pups was obtained using RIPA lysis buffer (Santa Cruz Biotechnology) with further centrifugation for 30 min at 14,000*g* at 4 °C. The supernatant was collected, and the protein concentration was determined using a detergent compatible assay (Bio-Rad). Equal amounts of protein were loaded on an SDS-PAGE gel. After being electrophoresed and transferred to a nitrocellulose membrane, the membrane was blocked and incubated with the primary antibody at 4 °C overnight. The primary antibodies included: rabbit anti-TET2 (ABE 364, EMD), rabbit anti-NF-κB p65 (8242, CST), rabbit anti-acetyl-p65 (ab19870, abcam), mouse anti-HDAC3 (3949 s, CST), mouse monoclonal anti-β-actin (A1978, Sigma-Aldrich), or rabbit polyclonal anti-GAPDH antibody (ab9485, Abcam). Nitrocellulose membranes were incubated with secondary antibodies (Santa Cruz Biotechnology) at room temperature for 1 h. Immunoblots were then probed with an ECL Plus chemiluminescence reagent kit (32132, Fisher Scientific) and visualized with the imaging system (Bio-Rad, Versa Doc, model 4000). The images were analyzed using the NIH Image J software.

### Co-Immunoprecipitation

Co-immunoprecipitation (co-IP) was done using the Pierce co-IP kit (26149, Fisher Scientific) following the manufacturer’s protocol. Briefly, the rabbit anti-TET2 antibody (ab124297, Abcam) was first immobilized for 2 h using AminoLink Plus coupling resin. The resin was then washed and incubated with tissue lysate overnight. After incubation, the resin was again washed, and protein was eluted using elution buffer. The rabbit immunoglobin G (IgG) was used as a negative control. The pull-down samples were probed by Western blotting using specific primary antibodies against TET2, NF-κB p65, acetyl-p65, HDAC3 or GAPDH as described above, and horseradish peroxidase-conjugated secondary antibodies.

### Chromatin immunoprecipitation (ChIP)

ChIP assays were performed using the SimpleChIP (#57976, CST) according to the manufacturer’s instructions. Briefly, tissues were minced and fixed with 1.5% formaldehyde to crosslink and maintain the DNA/protein interactions. After the reactions were stopped with glycine, tissues were washed with PBS. Chromatin extracts were sonicated to produce DNA fragments between 200 and 1000 base pairs. Rabbit anti-acetyl-p65 antibody (ab19870, Abcam) or negative control normal Rabbit IgG was incubated with the chromatin extracts to precipitate the transcription factor/DNA complexes. Crosslinking was then reversed using a salt solution, and proteins were digested with proteinase K. The antibody-pulled chromatin extracts were then subjected to real-time quantitative PCR analysis using IL-1β primers [39] targeting p65 binding region (Table 1).

### Luciferase reporter gene assay

A segment of 3′ UTR of mouse TET2 mRNA harboring the potential target region of mature miR-210 was PCR amplified from mouse brain cDNA using the forward (5′- gagaccc**GCTAGC**gtgcttctgcttggtgtcaa) and the reverse (5′-gagaccc**GTCGAC**aagcatagaaaaatgcacacaga) primers, designed based on mouse TET2 mRNA sequence (GENBANK accession #: 214133). The primers contained artificial NheI (GCTAGC) and SalI (GTCGAC) sites in forward and reverse primers respectively to facilitate cloning. Subsequently, NheI-217 bp-SalI TET2-derived fragment was cloned between NheI (5′) and SalI (3′) sites in the pmirGLO luciferase vector (Promega) to generate pmirGLOXTET2X reporter construct. The sequence of the TET2 derived segment was confirmed by Sequencing (Retrogen). For validation of the miR-210 target in mouse TET2 3′UTR, PC12 cells were co-transfected with either pmirGLO control vector or pmirGLO-TET2 construct with multiple doses of miR210 mimics (miR-210) (Qiagen) or scrambled miRNA. The *Firefly* and *Renilla reniformis* luciferase activities in cell extracts were measured using luminometer 48 h after co-transfection. The *Firefly* luciferase activity was normalized to *Renilla reniformis* luciferase activity and expressed as relative to control pmirGLO activity (% control), as we previously described [32, 40].

### Statistical analysis

Data were expressed as mean ± standard error of the mean (SEM). All graphs in this study were generated with GraphPad Prism 5. In experiments related to animals, experimental number (*n*) represents pups from at least two different dams. Comparisons between two groups were analyzed using Student’s *t* test (unpaired, two-tailed), and multiple comparisons were analyzed using one-way ANOVA followed by Newman-Keuls post hoc test. A *p* value less than 0.05 was considered significant.

## Results

### HI upregulated miR-210, suppressed TET2 expression, and increased p65 acetylation level and binding activity at IL-1β promoter in the brain of mouse pups

**Fig. 1:**
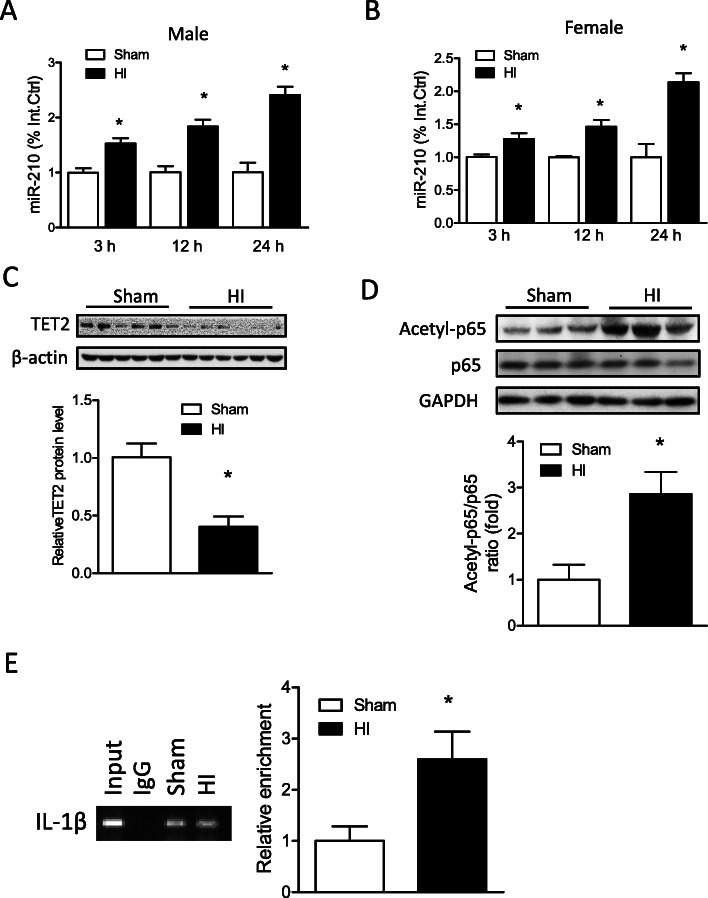
Effect of neonatal HI on miR-210 level, TET2 protein abundance, and p65 acetylation level and its DNA binding at IL-1β promoter in the mouse brain. HI brain injury was induced in postnatal day 7 (P7) mouse pups, the ipsilateral cerebral hemisphere of the brain was collected at 3, 12, and 24 h after HI. MiR-210 levels were detected in the ipsilateral hemisphere in Sham and HI in both male (**a**) and female pups (**b**) using qRT-PCR. *n* = 4 pups/group. *p < 0.05 vs Sham. At 12 h after HI, **c** TET2, **d** acetyl-p65, and total p65 protein levels were detected by Western blot. Data are expressed as mean ± SEM. *n* = 6 pups/group. *p < 0.05 vs Sham. **e** Chromatin immunoprecipitation (ChIP)-PCR assay of acetyl-p65 at the promoter of IL-1β. The enrichment of IL-1β was detected by qPCR. Data are expressed as mean ± SEM. *n* = 4 pups/group. *p < 0.05 vs Sham

Neonatal HI insult was introduced in P7 mouse pups with the ligation of the right common carotid artery followed by hypoxic (8% O_2_) treatment. HI insult significantly increased miR-210 levels in the brain in a time-dependent manner at 3, 12, and 24 h after the treatment in both male (Fig. 1a) and female pups (Fig. 1b). In addition, the HI treatment significantly decreased TET2 protein abundance (Fig. 1c) and increased the acetylation level of NF-κB subunit p65 (Fig. 1d) in the ipsilateral hemisphere of mouse brain at 12 h, compared with Sham. ChIP-PCR assay of acetyl-p65 showed that neonatal HI also significantly enhanced the binding of acetyl-p65 at the promoter of IL-1β (Fig. 1e).

### MiR-210 repressed TET2 expression in the neonatal brain

**Fig. 2:**
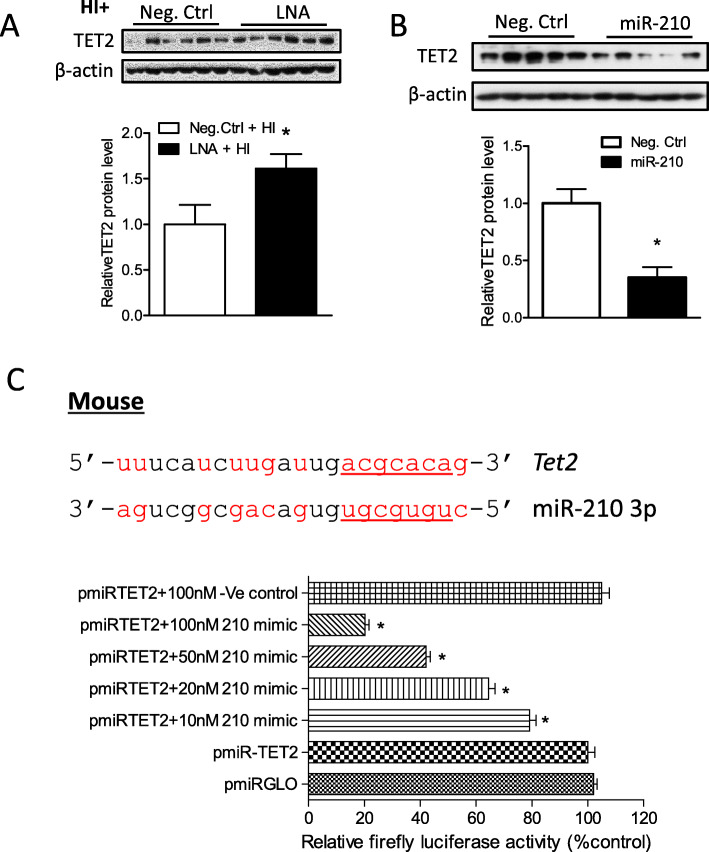
MiR-210 downregulated TET2 in the neonatal brain. **a** HI brain injury was induced in P7 mouse pups. Either miR-210-LNA (100 pmol) or scramble LNA (Neg. Ctrl) was administered into the ipsilateral hemisphere via i.c.v. injection 24 h prior to HI. **a** TET2 protein abundance was detected in the ipsilateral cerebral hemisphere of the brain 12 h after HI. Data are expressed as mean ± SEM. *n* = 6 pups/group. *p < 0.05 vs HI + Neg. Ctrl. **b** Either miR-210 mimic (100 pmol) or scramble (Neg. Ctrl) was administered into the right hemisphere of mouse brain via i.c.v. injection. TET2 protein level was detected by Western blot in the ipsilateral cerebral hemisphere of the brain 48 h after injection. Data are expressed as mean ± SEM. *n* = 5 pups/group. *p < 0.05 vs Neg. Ctrl. **c** Luciferase reporter gene assay of miR-210 targeting TET2 3′UTR. The diagram shows TET2 mRNA 3′UTR with the binding sites of miR-210 in mouse species. The pmirGLO plasmid inserted with TET2 3′UTR sequence containing putative miR-210 binding sites (pmiRTET2) was transfected into PC12 cells and was treated with either miR-210 mimics or scramble (Ve-control). Firefly and *Renilla reniformis* luciferase activities were measured in a luminometer using a dual-luciferase reporter assay system. *p < 0.05, treatment vs Ve control

To determine the role of miR-210 on TET2 expression after neonatal HI brain injury, either miR-210-LNA or its negative control was delivered via i.c.v. injection into the ipsilateral hemisphere 24 h before HI insult. At 12 h after HI, TET2 protein abundance was measured by Western blot. As shown in Fig. 2a, miR-210-LNA significantly rescued TET2 protein abundance, compared with the negative control (Neg. Ctrl). We then delivered the miR-210 mimic or negative control into the neonatal brain via i.c.v. injection. The results demonstrated that miR-210 mimic significantly decreased TET2 protein abundance (Fig. 2b) in the brain 48 h after the treatment. By searching the database of the predication of miR targets (TargetScan 7.1), we found that miR-210 has putative binding target sequences at the 3′ UTR of mouse TET2 transcript (Fig. 2c, upper panel). We thus performed a luciferase reporter gene assay to examine whether TET2 transcript was a direct target of miR-210. Using PC12 cells co-transfected with pmiRTE.T2 and multiple doses of miR-210 mimic or its negative control, we found that miR-210 mimic, but not its negative control, dose-dependently decreased luciferase activity (Fig. 2c, lower panel).

### TET2-HDAC3 interaction regulated DNA binding activity of NF-κB p65 at IL-1β gene promoter in the brain of naïve mouse pups

**Fig. 3:**
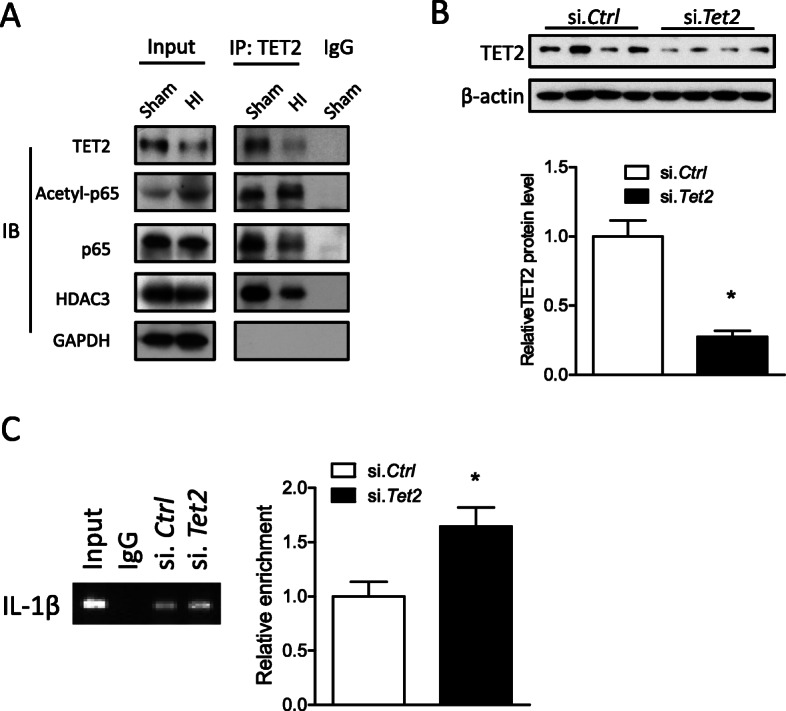
TET2 interacting with HDAC3 regulated p65 acetylation at IL-1β promoter. **a** HI brain injury was induced in mouse pups, and the ipsilateral cerebral hemisphere of the brain was collected 12 h after HI. Co-immunoprecipitation was applied using a specific antibody against TET2. The specific binding of TET2, p65, and HDAC3 was detected by Western blot using specific primary antibodies. Either TET2 siRNA (100 pmol; si. *Tet2*) or scramble (si. *Ctrl*) was administered into the right hemisphere of mouse brain via i.c.v. injection, and 48 h later, **b** TET2 protein level was detected by Western blot. Data are expressed as mean ± SEM. *n* = 4 pups/group. *p < 0.05 vs si. *Ctrl*. **c** Chromatin immunoprecipitation (ChIP)-PCR assay of acetyl-p65 at the promoter of IL-1β. The enrichment of IL-1β was detected by qPCR. Data are expressed as mean ± SEM. *n* = 5–6 pups/group. *p < 0.05 vs Sham

Co-immunoprecipitation (co-IP) using a specific antibody against TET2 revealed that TET2 interacted with p65 and HDAC3 (Fig. 3a). Furthermore, either TET2 siRNA (si. *Tet2*) or scramble siRNA (si. *Ctrl*) was delivered into the brain of mouse pups via i.c.v. injection. TET2 protein abundance (Fig. 3b) was significantly reduced 48 h after si. *Tet2* injection, compared with si. *Ctrl*. Of importance, ChIP-PCR assay using a specific antibody against acetyl-p65 demonstrated that TET2 knockdown significantly enhanced the binding of acetyl-p65 at the promoter of IL-1β gene in the brain of mouse pups (Fig. 3c).

### TET2 knockdown increased the acetylation level of NF-κB p65 and the expression of proinflammatory cytokines and chemokines after HI

**Fig. 4:**
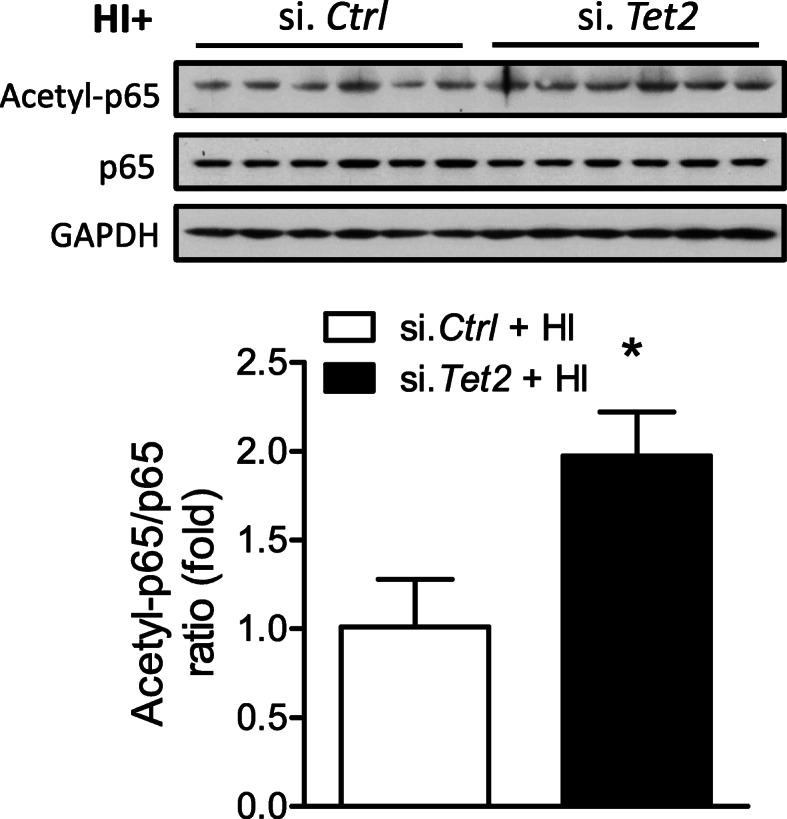
TET2 knockdown augmented acetyl-p65 level after neonatal HI. Either TET2 siRNA (100 pmol; si. *Tet2*) or scramble (si. *Ctrl*) was administered into the right hemisphere of both male and female mouse brain via i.c.v. injection, neonatal HI was induced 48 h after siRNA injection, and the ipsilateral cerebral hemisphere of the brain was collected 12 h after HI. Acetyl-p65 and total p65 protein levels were detected by Western blot. Data are expressed as mean ± SEM. *n* = 6 pups/group. *p < 0.05 vs Sham

**Fig. 5:**
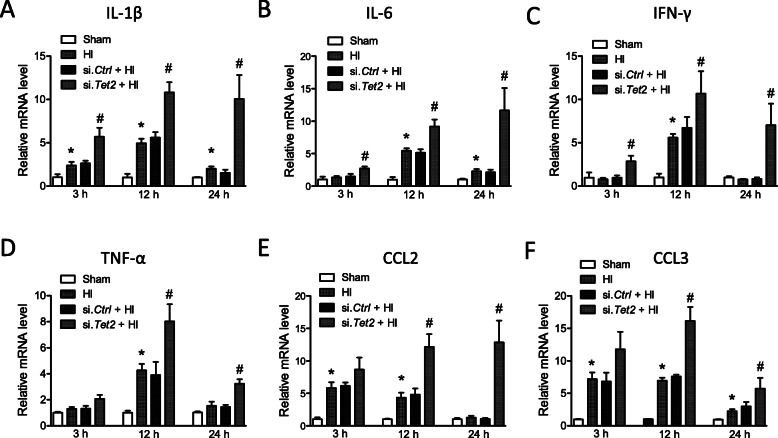
TET2 knockdown augmented pro-inflammatory cytokine levels after neonatal HI. The ipsilateral cerebral hemisphere of the brain was collected 3, 12, and 24 h after HI. qRT-PCR was performed for **a** IL-1 β, **b** IL-6, **c** IFN δ, **d** TNF α, **e** CCL2, and **f** CCL3. Data are expressed as mean ± SEM. *n* = 4 pups/group. *p < 0.05 vs Sham; ^#^*p* < 0.05 vs si. *Ctrl* + HI

To further confirm that TET2 regulates p65 acetylation, HI insult was introduced to mouse pups after either si. *Tet2* or si. *Ctrl* injection. The result of Western blot showed that si. *Tet2*, compared with si. *Ctrl*, significantly increased the acetylation level of p65 after HI (Fig. 4). To reveal the effect of TET2 on the expression of pro-inflammatory cytokines, mRNA levels of tumor necrosis factor alpha (TNF-α), IL-1β, interleukin-6 (IL-6), interferon gamma (IFN-γ), and C-C motif chemokine ligand 2 (CCL2) and 3 (CCL3) in the ipsilateral hemisphere were determined at 3, 12, and 24 h after neonatal HI (Fig. 5). The levels of IL-1β (Fig. 5a) and CCL3 (Fig. 5f) were significantly increased at all time-points after HI, compared with Sham. The levels of IL-6 (Fig. 5b) were significantly upregulated at 12 and 24 h, while IFN-γ and TNF-α were upregulated only at 12 h after HI. CCL2 (Fig. 5e) was significantly increased at 3 and 12 h, but not 24 h after HI. Of importance, TET2 knockdown significantly enhanced the expression of IL-1β, IL-6, and IFN-γ, and CCL3 at all time-points, while TNF-α, CCL2, and CCL3 only at 12 and 24 h after HI, compared with si. *Ctrl* (Fig. 5).

### TET2 knockdown exacerbated neonatal HI brain injury

**Fig. 6:**
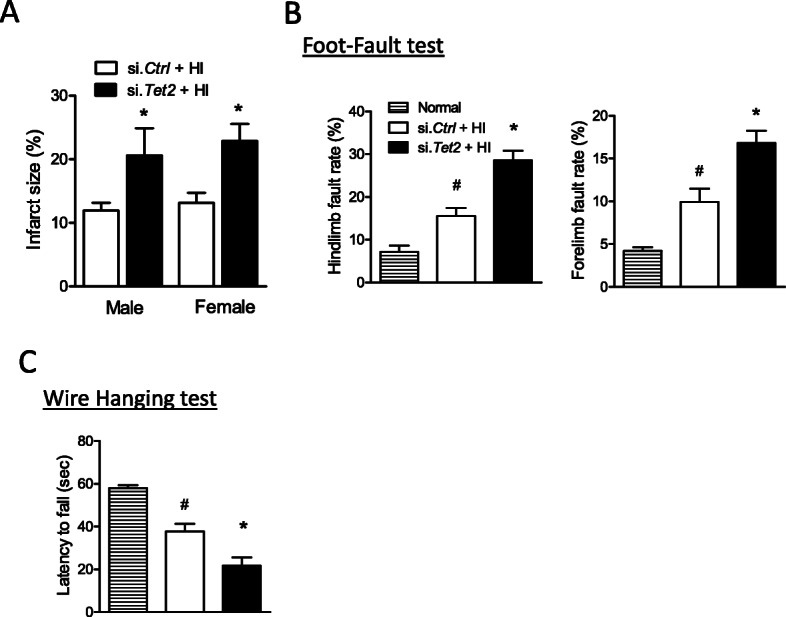
TET2 knockdown exacerbated neonatal HI brain injury. Either TET2 siRNA (100 pmol; si. *Tet2*) or scramble (si. *Ctrl*) was administered into the right hemisphere of both male and female mouse brain via i.c.v. injection, neonatal HI was induced 48 h after si.*Tet2* injection, and **a** brain infarct size was measured by TTC staining 48 h after HI. Data are expressed as mean ± SEM. *n* = 10 pups/group in si. *Ctrl*; *n* = 7–10 pups/group in si. *Tet2*. Two-tailed Student’s *t* test. **b** foot-fault test and **c** wire hanging test were performed 2 weeks after neonatal HI. Data are expressed as mean ± SEM. *n* = 6–9 pups/group in normal; *n* = 7–11 pups/group in si. *Ctrl*; *n* = 9–11 pups/group in si. *Tet2*. *p < 0.05 vs si. *Ctrl*. ^#^*p* < 0.05 vs normal

Mouse pups were subjected to HI with prior treatments of either si. *Tet2* or si. *Ctrl*, and brain infarct size was examined by TTC staining. Of note, a significant increase of 72% in brain infarct size in males and 74% in females were observed in the si. *Tet2*-treated animals 48 h after HI, compared with si. *Ctrl* (Fig. 6a), revealing a detrimental effect of TET2 downregulation on the neonatal brain. In addition, the neurological function was evaluated 2 weeks after HI insult. In the foot-fault test (Fig. 6b), animals with TET2 knockdown showed a significant increase in both hindlimb and forelimb fault rate induced by HI. Moreover, TET2 knockdown also significantly increased the latency to fall in the wire hanging test after HI, compared with si. *Ctrl* (Fig. 6c).

### TET2 knockdown counteracted the effect of miR-210 inhibition on inflammatory response in neonatal HI brain injury and in BV2 microglia cell line in vitro

**Fig. 7:**
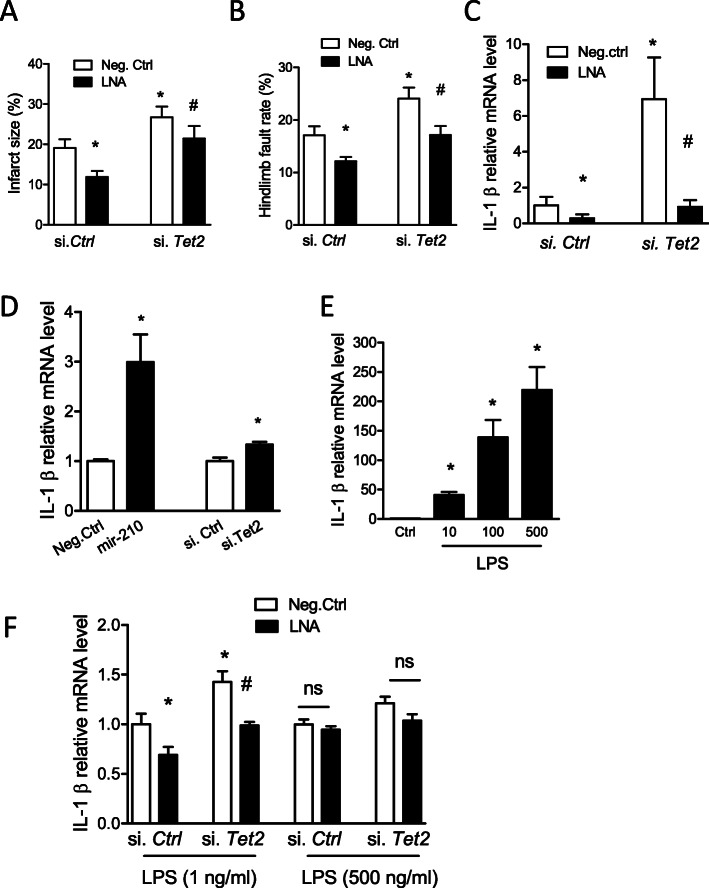
TET2 knockdown counteracted the effect of miR-210 inhibition on inflammatory response in neonatal HI brain injury and in BV2 microglia cell line in vitro. Either TET2 siRNA (100 pmol; si. *Tet2*) or scramble (si. *Ctrl*) was administered into the right hemisphere of both male and female mouse brain via i.c.v. injection, neonatal HI was induced 48 h after si. *Tet2* injection. Four hours after HI, either miR-210-LNA (100 pmol) or scramble LNA (Neg. Ctrl) was administered into the ipsilateral hemisphere of mouse brain via i.c.v. **a** Brain infarct size was measured by TTC staining 48 h after HI. Data are expressed as mean ± SEM. *n* = 8–9 pups/group. *p < 0.05 vs Neg. Ctrl-treated si. *Ctrl*. ^#^*p* < 0.05 vs miR-210-LNA-treated si. *Ctrl*. **b** The foot-fault test was performed 2 weeks after neonatal HI. Data are expressed as mean ± SEM. *n* = 9–12 pups/group in Neg. Ctrl-treated groups; *n* = 14–15 pups/group in LNA-treated groups. *p < 0.05 vs Neg. Ctrl-treated si. *Ctrl*. ^#^*p* < 0.05 vs miR-210-LNA-treated si. *Ctrl*. **c** The ipsilateral cerebral hemisphere of the brain was collected 24 h after HI. The qRT-PCR was performed for IL-1β transcript. Data are expressed as mean ± SEM. *n* = 4–5 pups/group. **d** BV2 cells were transfected with miR-210 mimic, si. *Tet2* or controls for 24 h. The qRT-PCR was performed for IL-1β transcript. Data are presented as the mean ± SEM. *n* = 3. **e** BV2 cells were stimulated with multiple doses of LPS for 6 h. The qRT-PCR was performed for IL-1β transcript. Data are presented as the mean ± SEM. *n* = 3. **e** BV2 cells were transfected with either miR-210-LNA or Neg. Ctrl in combination with si. *Tet2* or si. *Ctrl* for 24 h followed by LPS stimulation (1 ng/ml or 500 ng/ml) for 6 h. The qRT-PCR was performed for IL-1β transcript. Data are presented as the mean ± SEM. *n* = 6 in groups with 1 ng/ml LPS; *n* = 3 in groups with 500 ng/ml LPS. *p < 0.05 vs Neg. Ctrl-treated si. *Ctrl*. ^#^*p* < 0.05 vs miR-210-LNA-treated si. *Ctrl*. ns, not significant

To examine whether and to what extent TET2 mediates the effect of miR-210 in neonatal HI brain, mouse pups were treated with either si. *Tet2* or si. *Ctrl* 48 h prior to HI insult. Four hours after HI, either miR-210-LNA or scramble LNA (Neg. Ctrl) was delivered into the ipsilateral hemisphere of the brain via i.c.v. injection, and brain infarction was examined by TTC staining 48 h later. In animals pretreated with si. *Ctrl*, miR-210-LNA significantly reduced brain infarct size, compared with Neg. Ctrl. In contrast, in animals pretreated with si. *Tet2*, there was a lack of significant neuroprotective effect of miR-210-LNA in the neonatal HI brain injury (Fig. 7a). The foot-fault test was conducted 2 weeks after HI insult. The result showed that miR-210-LNA significantly reduced hindlimb fault rate in animals pretreated with si. *Ctrl*, compared with Neg. Ctrl, which was counteracted in animals pretreated with si. *Tet2* after HI insult (Fig. 7b). In addition, miR-210-LNA also significantly reduced the expression of key cytokine IL-1β in si. *Ctrl* pretreated animals, compared with Neg. Ctrl. Conversely, si. *Tet2* pretreatment significantly eliminates the effect of miR-210-LNA on IL-1β expression after HI insult, compared with si. *Ctrl* (Fig. 7c).

Our previous study has demonstrated that miR-210-LNA reduced LPS-stimulated upregulation of pro-inflammatory cytokines in rat microglia [41]. TET2 has been reported to regulate the expression of pro-inflammatory cytokines in macrophage/microglia [42–45]. We then test the role of miR210-TET2 axis in the regulation of inflammatory response in BV2 microglia. The result showed that transfection of either miR-210 mimic or TET2 siRNAs significantly upregulated the expression of IL-1β mRNA in BV2 microglia by about 3- and 1.3-folds, respectively (Fig. 7d). To optimize the doses of LPS treatment, BV2 microglia were stimulated with multiple doses of LPS (0, 10, 100, or 500 ng/ml) for 6 h. The result showed that LPS dose-dependently increased the expression of IL-1β in BV2 cells (Fig. 7e). Then, BV2 microglia were transfected with either miR-210-LNA or Neg. Ctrl in combination with si. *Tet2* or si. *Ctrl* for 24 h followed by LPS stimulation. The result showed that in BV2 microglia with low dose (1 ng/ml), but not high dose (500 ng/ml) LPS treatment, miR-210-LNA significantly reduced the expression of IL-1β in si. *Ctrl* transfected BV2 microglia, compared with Neg. Ctrl, which was reversed by si. *Tet2* transfection (Fig. 7f).

## Discussion

The inflammatory response occurs rapidly after HI onset and can last for several weeks, which is a major contributor to the brain injury in the immature brain following HI insult [16, 17]. Mounting evidence suggests that alleviation of early phase of inflammation confers neuroprotective effects after neonatal HI brain injury [46–49]. Our previous studies demonstrated that HI upregulated brain miR-210 in rat pups, and inhibition of brain miR-210 provided neuroprotection against neonatal HI brain injury [32, 33]. Thus, in the present study, we sought to investigate the underlying mechanism by which TET2 is negatively regulated by miR-210 and contributes to brain pro-inflammatory response after neonatal HI in mouse pups. We identified that HI upregulated brain miR-210 level in mouse pups the same as in rat pups [32]. Neonatal HI insult led to downregulation of TET2 levels and increase of acetyl-p65 binding at the promoter of IL-1β gene, a key marker of pro-inflammatory response after brain injury. This consequence following HI was potentially ascribed to the upregulation of miR-210, in that our in vivo and in vitro data showed that TET2 was a direct target of miR-210. We then determined the role of TET2 in neonatal HI-induced pro-inflammation and found that TET2 suppressed the DNA binding capacity of NF-κB p65 at IL-1β promoter in conjunction with HDAC3, a co-repressor of gene transcription. Moreover, TET2 knockdown augmented pro-inflammatory cytokine expression after neonatal HI. Regarding the functionality of TET2 in the neonatal brain, we demonstrated that knockdown of TET2 increased brain infarct size and exacerbated neurological deficits after neonatal HI, which reversed the neuroprotection of miR-210 inhibition in HI-induced brain injury. In addition, TET2 knockdown also counteracted the effect of miR-210 inhibition on HI-induced brain pro-inflammation. Finally, using BV2 mouse microglia cell line, we provided evidence that the miR-210-TET2 axis regulated pro-inflammatory response in microglia.

Previously, we determined that inhibition of brain miR-210 suppressed post-stroke inflammatory reaction in an ischemic brain injury model in adult mice [31]. This finding implicates that the detrimental effect of miR-210 on the neonatal brain [32] may attribute to its engagement in regulating pro-inflammatory response after HI. However, the downstream effector molecule of miR-210 in regulating neuroinflammation remains elusive. The present study identified TET2, a regulator of pro-inflammatory cytokine expression [42, 45], as a novel target of miR-210. Basically, miRs silence gene expression by binding to the 3′UTR of the transcript via their seed sequences at 5′ ends (nucleotides 2–8), resulting in transcript degradation or translational inhibition of the target genes. By bioinformatics analysis (TargetScan 7.1), we found that the 3′UTR of TET2 mRNA contains the highly-conserved binding sequences for miR-210 across species including human, rat, and mouse. We validated the result of bioinformatics analysis in in vivo experiment and found that injection of miR-210 mimic into the brain of mouse pups significantly reduced TET2 protein abundance, whereas inhibition of miR-210 rescued TET2 protein level after neonatal HI brain injury. Furthermore, the result of luciferase reporter gene assay revealed that miR-210 dose-dependently decreased luciferase activity in PC12 cells co-transfected with plasmid pmiRTET2, confirming that TET2 transcript is indeed a direct target of miR-210.

TET2 is a member of the ten eleven translocation (TET) methylcytosine dioxygenase family, which catalyzes the conversion of 5-methylcytosine (5mC) to 5-hydroxymethylcytosine (5hmC) in the mammalian genome and promotes DNA demethylation [50–52]. Recent studies documented that TET2 is engaged in the repression of the immune system [53, 54] and inflammation of macrophage [43, 45, 55–57]. TET2 insufficiency increased mRNA expression of IL-1β, IL-6, and Arginase1, indicating that TET2 can restrain macrophage-mediated inflammation [56]. It has been demonstrated that TET2 protein regulates transcription of pro-inflammatory cytokines by recruiting histone modifiers, such as HDACs, which is independent of TET2 catalytic activity [42, 45]. For example, TET2 recruits HDAC2 and mediates the repression of transcription of IL-6 and IL-1β via histone deacetylation [42]. In addition to the histone, HDACs can also regulate acetylation levels of non-histone transcription factors, thus modulating the transcription switch of target genes [58–60]. NF-κB subunit p65, the key transcription factor of early processes of immune and inflammatory responses, is a non-histone substrate of HDAC3, but not other HDACs [61]. The HDAC3-mediated deacetylation of p65 increases the binding with IκBα and enhances IκBα-dependent nuclear export, thus leading to turn-off of NF-κB transcriptional response [60, 62, 63]. Inspired by these findings, we hypothesize that TET2 recruits HDAC3 leading to p65 deacetylation and thus downregulates the transcription of pro-inflammatory cytokine genes. To test this hypothesis, we conducted co-immunoprecipitation and found the conjunction of TET2, HDAC3, and NF-κB p65 in the brain of mouse pups in both sham and HI groups. Furthermore, we injected TET2 siRNA into the neonatal brain in order to examine the TET2-HDAC3 co-repressor complex in regulating p65 acetylation. As we expected, silencing TET2 reduced the binding of acetyl-p65 at IL-1β promoter in the brain of naïve pups. In addition, it enhanced the upregulation of acetylation level of p65 after HI insult, compared with *si. Ctrl*-treated pups. These data demonstrate that TET2 regulates NF-κB p65 transcriptional activity in the expression of cytokine genes. Of importance, our result revealed that TET2 knockdown time-dependently upregulated the transcripts of a group of pro-inflammatory cytokines.

Next, we investigated the function of TET2 in the neonatal brain after HI insult and determined its innate protective role in the immature brain. We found that TET2 knockdown significantly increased brain infarct size and worsen the sensorimotor functions after neonatal HI, which is in agreement with the previous report that TET2 deficiency exacerbated infarct volume after ischemic brain injury in adult mice [64]. The finding that TET2 knockdown counteracted the neuroprotective effect of miR-210 inhibition is of great interest and provides functional evidence that TET2 is a downstream mechanism of miR-210 in the regulation of neuroinflammation and brain injury in neonatal HIE. Our previous study reported that the miR-210 mimic treatment upregulated the expression of pro-inflammatory cytokines in rat microglia [41], thus raising an interesting question, as an inflammation suppresso r[42, 56], whether TET2 mediates the effect of miR-210 on pro-inflammatory response in microglia. Using BV2 microglia, our results showed that TET2 knockdown significantly increased the expression of IL-1β mRNA similar to miR-210 mimic. Moreover, TET2 siRNAs also exacerbated low dose LPS stimulated IL-1β expression and reversed the effect of miR-210 inhibitor (LNA) in BV2 microglia. These findings suggested an orchestrator role of the miR-210-TET2 axis in pro-inflammatory response in microglia. Given that TET2 siRNA results in downregulation of TET2 protein abundance in the brain, we cannot rule out the potential effect of TET2 on the expression of pro-inflammatory cytokine through epigenetic modification. It is possible that the detrimental effect of TET2 knockdown on the neonatal brain is a potential consequence of multiple mechanisms regulated by TET2.

## Conclusion

In conclusion, the present study demonstrates that TET2 is negatively regulated by miR-210 and mediates inflammatory response after neonatal HI brain injury in mice. We determine that miR-210 directly binds to the 3′UTR of TET2 transcript. TET2 in conjunction with HDAC3 regulates the acetylation of NF-κB subunit p65, thus affecting the transcriptional activity of NF-κB on the expression of pro-inflammatory cytokines. Regarding the function of TET2 in the neonatal brain, we find that knocking down TET2 worsens neonatal HI-induced brain infarct and neurological deficits and reverses the neuroprotective effect of miR-210 inhibition. In addition, in vitro results suggest that the miR-210-TET2 axis regulates pro-inflammatory response in microglia. These findings identify the miR-210-TET2 axis in a molecular level and uncover a causative role of this axis in HI-induced inflammatory response in the neonatal brain, and substantiate the mechanism underpinning the neuroprotective effect of miR-210-LNA in neonatal HI brain injury.

## Data Availability

The datasets during and/or analyzed during the current study available from the corresponding authors on reasonable request.

## Abbreviations

3′UTR: 3′ Untranslated region
5mC: 5-Methylcytosine
5hmC: 5-Hydroxymethylcytosine
CCL2: C-C motif chemokine ligand 2
CCL3: C-C motif chemokine ligand 3
ChIP: Chromatin immunoprecipitation
co-IP: Co-immunoprecipitation
HDAC3: Histone deacetylase 3
HIE: Hypoxic-ischemic encephalopathy
IFN-γ: Interferon gamma
i.c.v.: Intracerebroventricular
IgG: Immunoglobin G
IL-6: Interleukin-6
IL-1β: Interleukin-1 beta
LNA: Locked nucleic acid oligonucleotides
LPS: Liposaccharide
miRs: MicroRNAs
SEM: Standard error of the mean
TET2: Ten eleven translocation methylcytosine dioxygenase 2
TNF-α: Tumor necrosis factor alpha
TTC: 2, 3, 5-Triphenyltetrazolium chloride monohydrate

## References

[1] Volpe JJ (2001). Perinatal brain injury: from pathogenesis to neuroprotection. Ment Retard Dev Disabil Res Rev.

[2] Fatemi A, Wilson MA, Johnston MV (2009). Hypoxic-ischemic encephalopathy in the term infant. Clin Perinatol.

[3] Vannucci RC (2000). Hypoxic-ischemic encephalopathy. Am J Perinatol.

[4] Lee AC, Kozuki N, Blencowe H, Vos T, Bahalim A, Darmstadt GL, Niermeyer S, Ellis M, Robertson NJ, Cousens S, Lawn JE (2013). Intrapartum-related neonatal encephalopathy incidence and impairment at regional and global levels for 2010 with trends from 1990. Pediatr Res.

[5] Graham EM, Ruis KA, Hartman AL, Northington FJ, Fox HE (2008). A systematic review of the role of intrapartum hypoxia-ischemia in the causation of neonatal encephalopathy. Am J Obstet Gynecol.

[6] Herrera-Marschitz M, Neira-Pena T, Rojas-Mancilla E, Espina-Marchant P, Esmar D, Perez R, Munoz V, Gutierrez-Hernandez M, Rivera B, Simola N (2014). Perinatal asphyxia: CNS development and deficits with delayed onset. Front Neurosci.

[7] Vannucci RC, Connor JR, Mauger DT, Palmer C, Smith MB, Towfighi J, Vannucci SJ (1999). Rat model of perinatal hypoxic-ischemic brain damage. J Neurosci Res.

[8] Koenigsberger MR (2000). Advances in neonatal neurology: 1950-2000. Rev Neurol.

[9] Zanelli G, Petrarca M, Cappa P, Castelli E, Berthoz A (2009). Reorientation ability of adults and healthy children submitted to whole body horizontal rotations. Cogn Process.

[10] Perlman JM, Wyllie J, Kattwinkel J, Atkins DL, Chameides L, Goldsmith JP, Guinsburg R, Hazinski MF, Morley C, Richmond S (2010). Part 11: Neonatal resuscitation: 2010 International Consensus on Cardiopulmonary Resuscitation and Emergency Cardiovascular Care Science With Treatment Recommendations. Circulation.

[11] Sabir H, Scull-Brown E, Liu X, Thoresen M (2012). Immediate hypothermia is not neuroprotective after severe hypoxia-ischemia and is deleterious when delayed by 12 hours in neonatal rats. Stroke.

[12] Papile LA, Baley JE, Benitz W, Cummings J, Carlo WA, Eichenwald E, Kumar P, Polin RA, Committee on F, Newborn (2014). Hypothermia and neonatal encephalopathy. Pediatrics.

[13] Azzopardi DV, Strohm B, Edwards AD, Dyet L, Halliday HL, Juszczak E, Kapellou O, Levene M, Marlow N, Porter E (2009). Moderate hypothermia to treat perinatal asphyxial encephalopathy. N Engl J Med.

[14] Liu F, McCullough LD (2013). Inflammatory responses in hypoxic ischemic encephalopathy. Acta Pharmacol Sin.

[15] Al Mamun A, Yu H, Romana S, Liu F (2018). Inflammatory responses are sex specific in chronic hypoxic-ischemic encephalopathy. Cell Transplant.

[16] Li B, Concepcion K, Meng X, Zhang L (2017). Brain-immune interactions in perinatal hypoxic-ischemic brain injury. Prog Neurobiol.

[17] Hagberg H, Mallard C, Ferriero DM, Vannucci SJ, Levison SW, Vexler ZS, Gressens P (2015). The role of inflammation in perinatal brain injury. Nat Rev Neurol.

[18] Algra SO, Groeneveld KM, Schadenberg AW, Haas F, Evens FC, Meerding J, Koenderman L, Jansen NJ, Prakken BJ (2013). Cerebral ischemia initiates an immediate innate immune response in neonates during cardiac surgery. J Neuroinflammation.

[19] Grether JK, Nelson KB (1997). Maternal infection and cerebral palsy in infants of normal birth weight. JAMA.

[20] Ziemka-Nalecz M, Jaworska J, Zalewska T (2017). Insights into the neuroinflammatory responses after neonatal hypoxia-ischemia. J Neuropathol Exp Neurol.

[21] Fleiss B, Nilsson MK, Blomgren K, Mallard C (2012). Neuroprotection by the histone deacetylase inhibitor trichostatin A in a model of lipopolysaccharide-sensitised neonatal hypoxic-ischaemic brain injury. J Neuroinflammation.

[22] Orrock JE, Panchapakesan K, Vezina G, Chang T, Harris K, Wang Y, Knoblach S, Massaro AN (2016). Association of brain injury and neonatal cytokine response during therapeutic hypothermia in newborns with hypoxic-ischemic encephalopathy. Pediatr Res.

[23] Silveira RC, Procianoy RS (2003). Interleukin-6 and tumor necrosis factor-alpha levels in plasma and cerebrospinal fluid of term newborn infants with hypoxic-ischemic encephalopathy. J Pediatr.

[24] Bartha AI, Foster-Barber A, Miller SP, Vigneron DB, Glidden DV, Barkovich AJ, Ferriero DM (2004). Neonatal encephalopathy: association of cytokines with MR spectroscopy and outcome. Pediatr Res.

[25] Liu Y, Zhang J, Han R, Liu H, Sun D, Liu X (2015). Downregulation of serum brain specific microRNA is associated with inflammation and infarct volume in acute ischemic stroke. J Clin Neurosci.

[26] Saraiva C, Talhada D, Rai A, Ferreira R, Ferreira L, Bernardino L, Ruscher K (2018). MicroRNA-124-loaded nanoparticles increase survival and neuronal differentiation of neural stem cells in vitro but do not contribute to stroke outcome in vivo. PLoS One.

[27] Xu X, Wen Z, Zhao N, Xu X, Wang F, Gao J, Jiang Y, Liu X (2017). MicroRNA-1906, a novel regulator of toll-like receptor 4, ameliorates ischemic injury after experimental stroke in mice. J Neurosci.

[28] Eyileten C, Wicik Z, De Rosa S, Mirowska-Guzel D, Soplinska A, Indolfi C, Jastrzebska-Kurkowska I, Czlonkowska A, Postula M. MicroRNAs as diagnostic and prognostic biomarkers in ischemic stroke-a comprehensive review and bioinformatic analysis. Cells. 2018;7(12):249. 10.3390/cells7120249 PMC6316722 30563269

[29] Esteller M (2011). Non-coding RNAs in human disease. Nat Rev Genet.

[30] Pasquinelli AE (2012). MicroRNAs and their targets: recognition, regulation and an emerging reciprocal relationship. Nat Rev Genet.

[31] Huang L, Ma Q, Li Y, Li B, Zhang L (2018). Inhibition of microRNA-210 suppresses pro-inflammatory response and reduces acute brain injury of ischemic stroke in mice. Exp Neurol.

[32] Ma Q, Dasgupta C, Li Y, Bajwa NM, Xiong F, Harding B, Hartman R, Zhang L (2016). Inhibition of microRNA-210 provides neuroprotection in hypoxic-ischemic brain injury in neonatal rats. Neurobiol Dis.

[33] Ma Q, Dasgupta C, Li Y, Huang L, Zhang L. MicroRNA-210 downregulates ISCU and induces mitochondrial dysfunction and neuronal death in neonatal hypoxic-ischemic brain injury. Mol Neurobiol. 2019;56:5608-25. 10.1007/s12035-019-1491-8 PMC6873808 30656514

[34] Nallamshetty S, Chan SY, Loscalzo J (2013). Hypoxia: a master regulator of microRNA biogenesis and activity. Free Radic Biol Med.

[35] Ma Q, Zhang L (2018). C-type natriuretic peptide functions as an innate neuroprotectant in neonatal hypoxic-ischemic brain injury in mouse via natriuretic peptide receptor 2. Exp Neurol.

[36] Sadakata T, Washida M, Iwayama Y, Shoji S, Sato Y, Ohkura T, Katoh-Semba R, Nakajima M, Sekine Y, Tanaka M (2007). Autistic-like phenotypes in Cadps2-knockout mice and aberrant CADPS2 splicing in autistic patients. J Clin Invest.

[37] Feather-Schussler DN, Ferguson TS. A battery of motor tests in a neonatal mouse model of cerebral palsy. J Vis Exp. 2016;117:53569. 10.3791/53569 PMC5226120 27842358

[38] Castelhano-Carlos MJ, Sousa N, Ohl F, Baumans V (2010). Identification methods in newborn C57BL/6 mice: a developmental and behavioural evaluation. Lab Anim.

[39] Scheibel M, Klein B, Merkle H, Schulz M, Fritsch R, Greten FR, Arkan MC, Schneider G, Schmid RM (2010). IkappaBbeta is an essential co-activator for LPS-induced IL-1beta transcription in vivo. J Exp Med.

[40] Dasgupta C, Chen M, Zhang H, Yang S, Zhang L (2012). Chronic hypoxia during gestation causes epigenetic repression of the estrogen receptor-alpha gene in ovine uterine arteries via heightened promoter methylation. Hypertension.

[41] Li B, Dasgupta C, Huang L, Meng X, Zhang L (2020). MiRNA-210 induces microglial activation and regulates microglia-mediated neuroinflammation in neonatal hypoxic-ischemic encephalopathy. Cell Mol Immunol.

[42] Zhang Q, Zhao K, Shen Q, Han Y, Gu Y, Li X, Zhao D, Liu Y, Wang C, Zhang X (2015). Tet2 is required to resolve inflammation by recruiting Hdac2 to specifically repress IL-6. Nature.

[43] Jiang S, Yan W, Wang SE, Baltimore D (2019). Dual mechanisms of posttranscriptional regulation of Tet2 by Let-7 microRNA in macrophages. Proc Natl Acad Sci U S A.

[44] Carrillo-Jimenez A, Deniz O, Niklison-Chirou MV, Ruiz R, Bezerra-Salomao K, Stratoulias V, Amouroux R, Yip PK, Vilalta A, Cheray M (2019). TET2 regulates the neuroinflammatory response in microglia. Cell Rep.

[45] Fuster JJ, MacLauchlan S, Zuriaga MA, Polackal MN, Ostriker AC, Chakraborty R, Wu CL, Sano S, Muralidharan S, Rius C (2017). Clonal hematopoiesis associated with TET2 deficiency accelerates atherosclerosis development in mice. Science.

[46] Welin AK, Svedin P, Lapatto R, Sultan B, Hagberg H, Gressens P, Kjellmer I, Mallard C (2007). Melatonin reduces inflammation and cell death in white matter in the mid-gestation fetal sheep following umbilical cord occlusion. Pediatr Res.

[47] Tsuji M, Wilson MA, Lange MS, Johnston MV (2004). Minocycline worsens hypoxic-ischemic brain injury in a neonatal mouse model. Exp Neurol.

[48] Tuor UI, Simone CS, Barks JD, Post M (1993). Dexamethasone prevents cerebral infarction without affecting cerebral blood flow in neonatal rats. Stroke.

[49] Felszeghy K, Banisadr G, Rostene W, Nyakas C, Haour F (2004). Dexamethasone downregulates chemokine receptor CXCR4 and exerts neuroprotection against hypoxia/ischemia-induced brain injury in neonatal rats. Neuroimmunomodulation.

[50] Zang S, Li J, Yang H, Zeng H, Han W, Zhang J, Lee M, Moczygemba M, Isgandarova S, Yang Y (2017). Mutations in 5-methylcytosine oxidase TET2 and RhoA cooperatively disrupt T cell homeostasis. J Clin Invest.

[51] Tahiliani M, Koh KP, Shen Y, Pastor WA, Bandukwala H, Brudno Y, Agarwal S, Iyer LM, Liu DR, Aravind L, Rao A (2009). Conversion of 5-methylcytosine to 5-hydroxymethylcytosine in mammalian DNA by MLL partner TET1. Science.

[52] He YF, Li BZ, Li Z, Liu P, Wang Y, Tang Q, Ding J, Jia Y, Chen Z, Li L (2011). Tet-mediated formation of 5-carboxylcytosine and its excision by TDG in mammalian DNA. Science.

[53] Feng Y, Li X, Cassady K, Zou Z, Zhang X (2019). TET2 function in hematopoietic malignancies, immune regulation, and DNA repair. Front Oncol.

[54] Ichiyama K, Chen T, Wang X, Yan X, Kim BS, Tanaka S, Ndiaye-Lobry D, Deng Y, Zou Y, Zheng P (2015). The methylcytosine dioxygenase Tet2 promotes DNA demethylation and activation of cytokine gene expression in T cells. Immunity.

[55] Liu Y, Peng W, Qu K, Lin X, Zeng Z, Chen J, Wei D, Wang Z (2018). TET2: a novel epigenetic regulator and potential intervention target for atherosclerosis. DNA Cell Biol.

[56] Cull AH, Snetsinger B, Buckstein R, Wells RA, Rauh MJ (2017). Tet2 restrains inflammatory gene expression in macrophages. Exp Hematol.

[57] Cai Z, Kotzin JJ, Ramdas B, Chen S, Nelanuthala S, Palam LR, Pandey R, Mali RS, Liu Y, Kelley MR (2018). Inhibition of inflammatory signaling in Tet2 mutant preleukemic cells mitigates stress-induced abnormalities and clonal hematopoiesis. Cell Stem Cell.

[58] Paz-Priel I, Houng S, Dooher J, Friedman AD (2011). C/EBPalpha and C/EBPalpha oncoproteins regulate nfkb1 and displace histone deacetylases from NF-kappaB p50 homodimers to induce NF-kappaB target genes. Blood.

[59] Gordon JW, Shaw JA, Kirshenbaum LA (2011). Multiple facets of NF-kappaB in the heart: to be or not to NF-kappaB. Circ Res.

[60] Calao M, Burny A, Quivy V, Dekoninck A, Van Lint C (2008). A pervasive role of histone acetyltransferases and deacetylases in an NF-kappaB-signaling code. Trends Biochem Sci.

[61] Kiernan R, Bres V, Ng RW, Coudart MP, El Messaoudi S, Sardet C, Jin DY, Emiliani S, Benkirane M (2003). Post-activation turn-off of NF-kappa B-dependent transcription is regulated by acetylation of p65. J Biol Chem.

[62] Chen L, Fischle W, Verdin E, Greene WC (2001). Duration of nuclear NF-kappaB action regulated by reversible acetylation. Science.

[63] Chen LF, Mu Y, Greene WC (2002). Acetylation of RelA at discrete sites regulates distinct nuclear functions of NF-kappaB. EMBO J.

[64] Miao Z, He Y, Xin N, Sun M, Chen L, Lin L, Li J, Kong J, Jin P, Xu X (2015). Altering 5-hydroxymethylcytosine modification impacts ischemic brain injury. Hum Mol Genet.

